# Synergistic Effect of Statins and Abiraterone Acetate on the Growth Inhibition of Neuroblastoma *via* Targeting Androgen Receptor

**DOI:** 10.3389/fonc.2021.595285

**Published:** 2021-05-10

**Authors:** Zengchun Hu, Chuandong Cheng, Yue Wang, Tianrui Chen, Junhong Tu, Chaoshi Niu, Rong Xing, Yang Wang, Yinghui Xu

**Affiliations:** ^1^ Dalian Medical University, Dalian, China; ^2^ Department of Neurosurgery, 2^nd^ Affiliated Hospital of Dalian Medical University, Dalian, China; ^3^ Anhui Provincial Hospital, Cheeloo College of Medicine, Shandong University, Jinan, China; ^4^ Division of Life Sciences and Medicine, Department of Neurosurgery, 1^st^ Affiliated Hospital of University of Science and Technology of China, Hefei, China; ^5^ Department of Pathophysiology, College of Basic Medical Sciences, Dalian Medical University, Dalian, China; ^6^ Department of Bone Tumor Surgery, Changzheng Hospital, Second Military Medical University, Shanghai, China; ^7^ Department of Neurosurgery, 1^st^ Affiliated Hospital of Dalian Medical University, Dalian, China

**Keywords:** statin, abiraterone acetate, synergistic effect, androgen receptor, neuroblastoma, SREBP cleavage activating protein

## Abstract

Neuroblastoma is the most common extracranial neuroendocrine tumor in childhood. Although many studies have tried to find effective treatments, there are still numerous limitations in current clinical targeted therapy. So, it is important to find new therapeutic targets and strategies from a new perspective. Our previous study reported that the androgen receptor (AR) promotes the growth of neuroblastoma *in vitro* and *in vivo*. Based on documentary investigation, we postulated that the AR–SCAP–SREBPs-CYP17/HMGCR axis may regulate cholesterol and androgens synthesis and form a positive enhancement loop promoting NB progression. Clinical samples and Oncomine database analysis proved the activation of AR–SCAP–SREBPs-CYP17/HMGCR axis in neuroblastoma. The combination of inhibitors of HMGCR (statins) and CYP17A1 (abiraterone acetate) showed synergistic effect that significantly inhibited the proliferation and migration with decreased expression of related genes detected *in vitro* and *in vivo* suggesting the dual-targeted therapy had the potential to inhibit the progression of neuroblastoma in spite of its MYCN status. This study provides new ideas for clinical treatment of neuroblastoma with efficacy and reduced toxicity.

## Introduction

Neuroblastoma (NB), a rare but the most common extracranial solid tumor in childhood, is an embryonic neuroendocrine tumor derived from the progenitor cells of sympathetic neural crest (1). In the United States, its incidence is about 1/7,000 of live births, and more than 650 cases are diagnosed with neuroblastoma every year (2). In patients with high-risk neuroblastoma, the therapies targeting PI3K/Akt/mTOR signaling have limitations. For instance, IBL‐302, a PIM/PI3K/mTOR triple kinase inhibitor, was recommended for MYCN-amplified NB treatment (3), whereas Periposine, an Akt inhibitor, was invalid for MYCN-amplified NB (4). Although the 5-year survival rate is about 50% at present (5), management options are so limited that innovative and effective approaches are necessary.

In the previous study, we found that the androgen receptor (AR) agonist, R1881, promoted the growth of NB *in vitro* and *in vivo* and the AR antagonists, MDV3100 and ARN509, significantly decreased the proliferation, migration, invasion, and sphere formation of NB cells cultured in hormone-free medium (6). The findings suggested that NB cells were able to produce androgens for the growth of themselves. Cholesterol is considered playing an important role in the development of some cancers. On one hand cholesterol is the precursor for sex hormones synthesis, while on the other it is an essential component of cell plasma membrane. Compared with normal cells, tumor cells need much more cholesterol in order to rapidly proliferate and synthesize new plasma membrane. The increased or activated cholesterol synthesis is related to the progression and aggravation of sarcoma, acute myeloid leukemia, melanoma (7), breast cancer (8), and prostate cancer (9). Therefore, targeting cholesterol synthesis pathway becomes promising. Some recent studies linked AR to cholesterol synthesis. In Neuro2a cells (N2a, mouse neuroblastoma cells), sterol regulatory element binding proteins (SREBPs) promote cholesterol synthesis *via* regulating 3-hydroxy-3-methylglutaryl-coenzyme A reductase (HMGCR), the rate limiting enzyme in cholesterol synthesis (10), on transcription level (11). Another research reported that AR/mTOR axis promoted the expression and activity of SREBP1, lead to androgen-dependent *de novo* synthesis of lipid and facilitated growth of prostate cancer (PCa) cells (12). High glucose concentration transports SREBP cleavage activating protein (SCAP) to Golgi matrix to activate SREBP (13), thus mature N-terminal fragment of SREBP enters nucleus to regulate HMGCR transcription (14). Hashimoto M. et al. proved that AR can increase the expression of SREBP2 target genes (9) and promote cholesterol synthesis through SCAP (15, 16). More than that, targeting SCAP inhibited the activity of SREBP and significantly reduced the growth of glioma cells in a nude mice model (14). Last but not least, Cytochrome P450 17A1 (CYP17A1) is the rate limiting enzyme in the synthesis of testosterone from cholesterol (17). The growth of glioma was inhibited by silencing *CYP17A1* gene which was reported transcriptionally regulated by SREBP (18).

In brief, we hypothesized that testosterone, produced under the control of CYP17A1, activated AR and then the transcription of *SCAP* was increased by AR (16) in NB cells. Next, SCAP promotes the maturation of SREBPs which increase the transcription of *HMGCR* and *cyp17a1* resulting in enhanced cholesterol synthesis and testosterone production, respectively. Finally, an ar-scap-srebp-cyp17A1/HMGCR positive enhancement loop forms and promotes the progress of NB.

Statins, inhibitors of HMGCR, are widely used in cholesterol lowering therapy. Simvastatin and fluvastatin, both showed the most common side effect of myopathy partly due to their lipophilic nature (19). Interestingly, statins’ anticancer utilities have been investigated in breast cancer, multiple myeloma and neurofibromatosis, lung cancer, metastatic colorectal cancer, and acute myeloid leukemia (20). Abiraterone acetate (AA, Zytiga), a CYP17A1 inhibitor, is the first-line treatment for metastatic castration resistant prostate cancer (mCRPC). Its most common adverse events include hypokalemia, fluid retention, and hypertension, but the combination of low-dose glucocorticoids can reduce the occurrence of these events to a large extent (21). Amazingly, combination use of one statin and AA showed a remarkable effect on the growth inhibition of NB cells. In the present research, we aimed at developing a new strategy to simultaneously inhibit the progression of NB and to reduce the side effects with combined use of statin and AA in lower doses.

## Materials and Methods

### Cell Culture and Human Tissue Samples

SK-N-BE (2) was obtained from Procell Life Science &Technology (Procell, Hubei, China); HL7702 was obtained from COBIOER (Cobioer Biosciences, Jiangsu, China); N2a and SH-SY5Y were kind gifts from Dr. Yuxian Shen (Anhui Medical University, Anhui, China); 10A, HUVEC and HepG2 were kind gifts from Dr. Kai Xue, Dr. Lei Shi, and Prof. Cong Li (Dalian Medical University, Liaoning, China), respectively; SK-N-BE (2) cell was maintained in Dulbecco’s modified Eagle’s medium (DMEM)/Ham’s F12 medium; HL7702 was maintained in RPMI 1640 medium; N2a,SH-SY5Y, and 10A cells were maintained in DMEM high glucose medium and HepG2 cells were maintained in Eagle’s Minimum Essential Medium (EMEM) in a humidified incubator with 5% CO_2_ at 37°C. The culture medium was supplemented with 10% fetal bovine serum FBS (AusGeneX), 100 U/ml penicillin, and 100 mg/ml streptomycin. By the end of the study, the passage number of any cell line mentioned above was below 20.

The formalin-fixed-paraffin-embedded tissue samples, including one neuroblastoma, one ganglioneuroblastoma, and four olfactory neuroblastoma tissues were obtained from the First Affiliated Hospital of University of Science and Technology of China (USTC); eight neuroblastoma, one ganglioneuroblastoma, three olfactory neuroblastoma, and six retinoblastoma samples were obtained from the Second Affiliated Hospital of Dalian Medical University (DMU). We collected all patient-derived specimens under protocols approved by the Institutional Review Boards of the Second Affiliated Hospital of DMU and the First Affiliated Hospital of USTC.

### Drugs and Antibodies

Simvastatin, fluvastatin, and rosuvastatin was purchased from MCE (Shanghai, China). Abiraterone acetate was obtained from Aladdin (Shanghai, China). Polyclonal anti-β-actin, polyclonal anti-Ki67, polyclonal anti-HMGCR, polyclonal anti-SREBP1, polyclonal anti-Ki67, and anti-AR (N-20) antibodies were obtained from Santa Cruz (TX, USA). Monoclonal anti- SCAP, Monoclonal anti-CYP17A1 were obtained from Proteintech (Hubei, China), Monoclonal anti-SREBP2 were obtained from Abcam (Shanghai, China).

### Cell Viability Assay

N2a, SH-SY5Y, SK-N-BE (2), 10A, HUVEC, HL7702 cells were plated in 96-well plates at 8,000 cells/well, respectively, in complete media in triplicate wells for each dose and cultured for 18 h. The neuroblastoma cell lines, N2a, SH-SY5Y, and SK-N-BE (2), and non-cancer cell lines, 10A, HUVEC, and HL7702, were treated with simvastatin and fluvastatin in a range of doses from 0 to 20 μM and with AA from 0 to 30 μM; N2a, SH-SY5Y SK-N-BE (2), were treated with rosuvastatin in a range of doses from 0 to 100 μM for 72 h. Statins dissolved in DMSO and AA dissolved in ethanol. After 72 h treatment, the medium was added 0.5 mg/ml MTT reagent and incubated at 37°C for 4 h. Subsequently, the supernatant was aspirated, and cells were lysed in 150 µl DMSO, then shaken 10 min at 37°C. The optical density (OD) was measured at 570 and 630 nm using a plate reader.

### Western Blots

Cell lysis, protein extraction, and immunoblotting were performed as described previously (22).

### RNA Expression Analysis

Total RNA was extracted from cell pellets or fresh tissues using the TRIzol reagent (Invitrogen, USA). RT-PCR was undertaken with TransScript First-Strand cDNA Synthesis SuperMix (Transgen Biotech, Beijing, China) and 2 × EasyTaq PCR SuperMix (cwBiotech, Beijing, China) according to the manufacturer’s instructions. The following primer pairs were used: SCAP (Forward; 5′ CCCCAGGCTATGACTTCAGC-3′) and (Reverse: 5′-CCAAGCT CCAGATGGAACCC-3′), SREBF1 (Forward: 5′-CTGTTCCTGTGTGACCTGCT-3′) and (Reverse: 5′-CATGTAGGAACACCCTCCGC-3′), SREBF2 (Forward: 5′-CTGGGAGACATCG ACGAGAT-3′) and (Reverse: 5′-GACCTGGGTGAATGACCGTT-3′), HMGCR (Forward: 5′- GTCATTCCAGCCAAGGTTGT-3′) and (Reverse: 5′-GGGACCACTTGCTTCCATTA-3′), CYP17A1 (Forward:5′-TTCAGCCGCACACCAACTAT-3′) and (Reverse: 5′-GGATTCAAG AAACGCTCAGGC-3′), ACTB (β-actin, Forward: GCTCGTCGTCGACAACGGCT) and (Reverse: CAAACATGATCTGGGTCATCTTCTCT).

### Wound Healing Assay

N2a, SH-SY5Y, and SK-N-BE (2) cells were cultured in six-well plates (5 × 10^5^cells/well) and incubated till they reached 90–100% confluence. The cells were then maintained in phenol red-free DMEM/DMEM-F12 with 2.5%FBS, in order to minimize the cell proliferation. A sterile 20 µl tip was used to create scratch wounds of the same width. The plates were then washed twice with phosphate-buffered saline (PBS) to remove the detached cells. Photos were taken at 0, 24, and 48 h, and the area covered by the cells enumerated the closure of the wounds. Each experiment was performed in triplicate.

### Immunohistochemistry

Immunohistological analysis was performed as previously described (23). The dilution and incubation with antibodies were as follows: anti-AR 1:250, anti-SCAP 1:100, anti-SREBP1 1:20, anti-SREBP2 1:100, anti-HMGCR 1:10, anti-CYP17A1 1:200, and anti-Ki67 1:250. The sections were then counterstained with hematoxylin.

### Tumor Xenograft Studies

Four weeks old BALB/c nude mice of both sexes were purchased from Vital River Laboratory Animal Technology Co., Ltd. (Beijing, China) and maintained under specific pathogen-free conditions. Male mice were castrated 5 days before cell inoculation. A total of 5 × 10^6^ SH-SY5Y cells were suspended in PBS to a final volume of 100 μl and injected subcutaneously into the flanks of a 6-week-old nude mouse. Then, all the mice were divided randomly into four groups: control group (Placebo, n = 7), simvastatin group (Sim, n = 6), abiraterone acetate group (AA, n = 6), and combination group (Com, n = 6) including three male mice in each group. Mice were then treated orally when the volume of the tumor reached 100 mm^3^ estimated with calipers as described previously (23) with drugs at the following doses and routes: simvastatin 20 mg/kg/day; AA 150 mg/kg/day; combined simvastatin 10 mg/kg/day with AA 75 mg/kg/day. Tumor was harvested after 10 days of dosing, then fixed half of a tumor in 4% buffered formaldehyde for IHC and the other half was stored −80°C for RT-PCR and Western Blots analysis.

### Ethics Approval

All mice experiments were carried out with ethical committee approval and met the standards required by the Dalian Medical University Animal Care and Use Committee guidelines.

### Bioinformatic Analysis of Gene Clusters

The study was based on a cohort of neuroblastoma patients described in the Oncomine database, analyzed the impact of two clusters of six genes (*AR*, *SCAP*, *SREBF1*, *SREBF2*, *HMGCR*, *CYP17A1*, or *LC3*, *ULK2*, *ATG8L*, *ATG12*, *ATG14*, *ATG21*) on the survival of NB patients. Data from patients aged 60 months or less (n = 133) were focused on for analysis. The index weight coefficient for grouping and 95% confidence interval were shown in **Supplementary Table S1**. Cox regression was used to calculate the regression coefficient of each cluster of six genes and calculate the risk score for each patient (**Supplementary Tables S1A–D**). By median ranking, the patients were divided into high- and low-risk groups. The risk score for each patient was derived by multiplying the expression level of a gene by its corresponding coefficient (risk score = sum of Cox coefficient of gene multiplied by expression value of gene) (24).

### Survival Analysis

All tests were carried out using SPSS (version 23.0; Chicago, USA). Kaplan-Meier curves were generated using GraphPad Prism 8.3. Comparisons between two cohorts were performed by the Log-Rank test.

### Quantification of Steroid Hormones

SH-SY5Y cells and HepG2 cells were seeded in 6 cm dishes in 50% confluency. The culture medium was discarded and washed three times with PBS 12 h after seeding. High glucose DMEM or EMEM (Hyclone) supplied with 5% charcoal-stripped fetal bovine serum (cFBS, Hyclone) for a hormone-free condition. Cell culture supernatant was centrifuged at 10,000 rpm and collected 72 h after cFBS treatment and stored at −80°C until shipping to BGI, Shenzhen (https://en.genomics.cn/en-medical-diagnosiss-Testingservices-Nutritionals.html), in dry ice. The sample pretreatment, LC-MS/MS analysis, and data processing were performed according to BGI’s standard procedures (25). The relative steroid hormone level of SH-SY5Y was calibrated with HepG2 cells.

### Data Analysis 

Statistical analysis was performed in GraphPad Prism Version 8 (GraphPad Software, San Diego, USA) statistical software. Data are presented as means ± standard division of at least three independent experiments. Data were analyzed by two-tailed Student’s t-test for comparisons between two groups, or one-way analysis of variance (ANOVA) with *post hoc* Bonferroni multiple comparison test for comparisons involving greater than two groups and p-values of p < 0.05 (∗), p < 0.01 (∗∗), p < 0.001 (∗∗∗), and p < 0.0001 (∗∗∗∗) were considered significant and highly significant, respectively.

## Results

### Neuroblastoma Is Characterized by the AR-SCAP-SREBPs-CYP17A1/HMGCR Axis Activation in Clinical Samples

**Figure 1:**
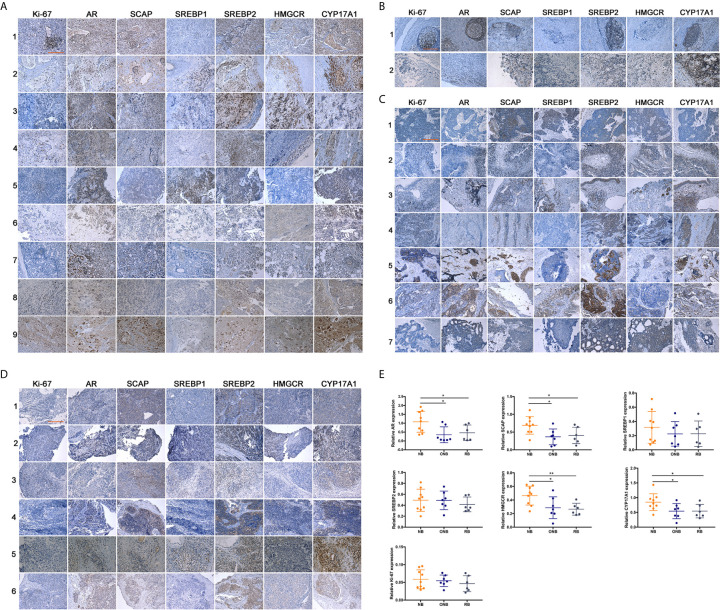
Expression of related proteins in clinical neuroblastic tumors analyzed by IHC. Images were visualized by inverted microscope. Scale bar = 250 μm. **(A)** Neuroblastoma samples, n = 9; **(B)** Ganglioneuroblastoma samples, n = 2; **(C)** Olfactory neuroblastoma samples, n = 7; **(D)** Retinoblastoma samples, n = 6; **(E)** Intensity of immunohistochemical staining was auto-calculated by Image-Pro Plus 6.0 to test positive rate, and quantitative map was plot by GraphPad Prism 7. The data are reported as mean ± SD (*P < 0.05, **P < 0.01).

Although the function of AR in neuroblastoma cells were investigated in the previous work, the expression of AR and other proteins in AR-SCAP-SREBPs-CYP17A1/HMGCR Axis had never been examined in clinical samples of neuroblastoma or other types of neuroblastic or neuroendocrine tumors. From 2010 to 2019, we collected clinical samples from NB (n = 9, retroperitoneal origin, mainly younger than 5 years old) (26), ganglioneuroblastoma (GNB, n = 2), olfactory neuroblastoma (ONB, n = 7), and retinoblastoma (RB, n = 6) patients in 1^st^ Affiliated Hospital of University of Science and Technology of Chima (USTC) and 2^nd^ Affiliated Hospital of Dalian Medical University (DMU) for IHC analysis (**Figures 1A–D**) to know the expression pattern of related proteins. Peripheral neuroblastic tumors are further classified based on their morphological features into NB, GNB, and ganglioneuroma (GN), known as the International Neuroblastoma Pathology Classification (INPC; Shimada system), which are relevant biologically and prognostically (27). Compared with ONB (**Figure 1C**) and RB (**Figure 1D**), the IHC staining of retroperitoneum-initiated NB showed significant higher (P < 0.05) expression of AR, SCAP, and especially the drug targets, HMGCR and CYP17A1 (**Figure 1E**). The result supported our hypothesis that AR signaling promoted the progression of neuroblastoma specifically. There was no significant difference in the intensity of SREBPs staining in the three types of neuroblastic tumors (**Figure 1E**). But in NB group SREBPs mostly localized to the nuclear area, whereas SREBPs staining appeared in the cytoplasm in ONB or RB group (**Supplementary Figure S1**), which indicated the activation of SREBPs in NB (28). The result of IHC showed that although the three neuroblastic tumors are related, NB is special for the activation of AR-SCAP-SREBPs-CYP17A1/HMGCR axis.

### Upregulation of the AR-SCAP-SREBP1/2-HMGCR/CYP17A1 Axis Is Associated With Poor Survival in Both Mycn-Amplified and Mycn-Non-Amplified NB

**Figure 2:**
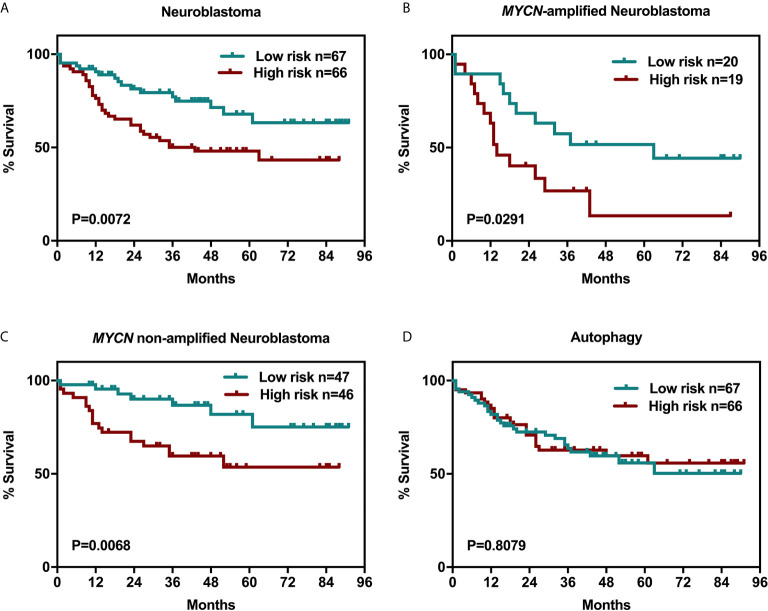
Six-related genes signature negatively correlated with overall survival in NB patients. Kaplan-Meier survival curves plotted to estimate the overall survival probabilities for the low-expression *vs* high-expression group. **(A)** overall survival of 133 NB patients grouped by the expression of a cluster of genes including *AR*, *SCAP*, *SREBF1*, *SREBF2*, *HMGCR*, and *CYP17A1*. **(B)** MYCN-amplified NB patients. **(C)** MYCN non-amplified NB patients. **(D)** overall survival of 133 NB patients grouped by the expression of a cluster of genes including *LC3*, *ULK2*, *GABARAPL1*(*Atg8L*), *Atg12*, *Atg14*, and *Atg21*.

It is still unknown that whether the expression of the six genes, *AR*, *SCAP*, *SREBF1*, *SREBF2*, *HMGCR*, and *CYP17A1* are closely associated with neuroblastoma patient survival. We examined clinical outcomes in the Oncomine Neuroblastoma Dataset of 133 patients who were divided into high- and low-expression groups (the grouping method was described in the *Material and Method* section) according to the simultaneous expression level of the six genes, the index weight coefficient, and 95% confidence interval were shown in **Supplementary Table S1**. Interestingly, upregulation of the six genes was related to unfavorable survival rate with statistical significance (**Figure 2A**, p = 0.0072) in spite of MYCN-amplification status (**Figures 2B, C**). Meanwhile, the risk scores of 133 patients including MYCN-amplified (n = 39) and MYCN non-amplified (n = 93) were calculated (**Supplementary Tables S1B, C**). With this method, we evaluated whether the expression of six autophagy-related genes (**Supplementary Table S1D**) was associated with the same 133 patients’ survival to exclude the possibility that a random cluster of six genes expression might significantly impact on the survival rate (**Figure 2D**).

### Synergy Between Statins and Abiraterone Acetate Against Neuroblastoma Cells *In Vitro*

**Figure 3:**
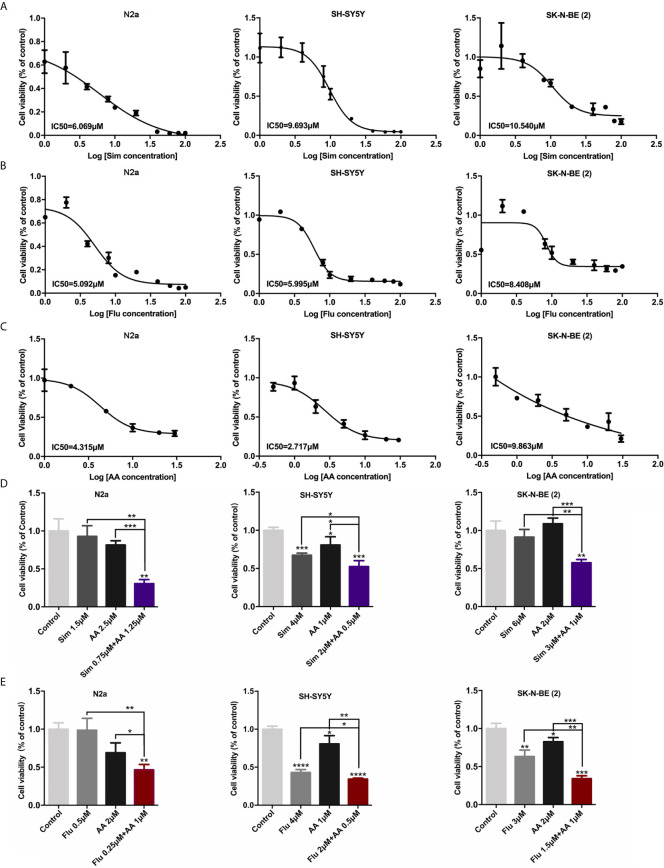
Statins and abiraterone acetate inhibited cell proliferation and showed synergistic effect. **(A–C)** N2a, SH-SY5Y, and SK-N-BE (2) were treated with Sim, Flu, or AA, the cell proliferation was detected by MTT assay after 72 h treatment. IC50 of cells was calculated using Graphpad prim 7. **(A, B)** Statins were dissolved in DMSO, and the final concentrations were 0, 1.0, 2.0, 4.0, 8.0, 10, 20 μM. **(C)** AA was dissolved in ethanol, the final concentrations were 0, 0.5, 1.0, 2.0, 5.0, 10, 20, 30 μM concentration gradient. **(D, E)** The effect of combination use of a statin and AA on cell viability was evaluated using MTT method. In the control group, 1 ‰ DMSO and 1 ‰ ethanol was added to the culture medium, 1 ‰ ethanol was added to the culture medium with statins alone, and 1 ‰ DMSO was added to the culture medium with AA alone. Half-dose of each single drug was used for the combination of two drugs. The difference was analyzed by GraphPad. Data are presented as mean ± standard division (SD) in three independent experiments. *p < 0.05, **p < 0.01, ***p < 0.001, ****p < 0.0001.

Based on the hypothesis mentioned above, statin or abiraterone acetate (AA) is supposed to show a growth inhibition effect on NB cells independently. As expected, statins and AA inhibited the proliferation of three NB cell lines, N2a, SH-SY5Y (both are *MYCN* non-amplified) and SK-N-BE (2) (*MYCN* amplified), in a dose-dependent manner (**Figures 3A–C**) analyzed with MTT method. Then we calculated the half-maximal inhibitory concentrations (IC50) of statins and AA in NB and normal cell lines at 72 h (**Table 1**). Surprisingly, compared with simvastatin and fluvastatin, rosuvastatin displayed a remarkable higher IC50 (**Supplementary Figure S2**). Moreover, to investigate if these drugs were toxic to non-cancer cell lines, the IC50 of human hepatocyte HL-7702, as well as human breast cell line 10A and endothelial cell line HUVEC was determined. All the three non-cancer cell lines showed less sensitive to simvastatin (**Table 1** and **Supplementary Figure S2**). Further, the efficacy of half-dose combination of a statin with AA on NB cells growth inhibition was compared with that of single treatment (**Figures 3D, E**). Evident synergistic effect was observed. For instance, the inhibition rate was about 10 and 20% with 1.5 μM simvastatin and 2.5 μM AA, respectively, while the inhibition rate was 70% with combined 0.75 μM simvastatin and 1.25 μM AA in N2a cells. But, astonishingly, the combination of drugs had limited effect on the growth of normal cells (**Supplementary Figure S3**). The results suggested that combined HMGCER and CYP17A1 inhibition synergistically restrained neuroblastoma proliferation and this strategy showed good safety.

**Table 1:** IC50 of statins and abiraterone acetate in NB and normal cell lines.

Drugs	IC50(μM)					
	NB Cell Lines			Normal Cell Lines		
	N2a	SH-SY5Y	SK-N-BE (2)	10A	HUVEC	HL7702
Simvastatin	6.069	9.693	10.54	26.66	14.58	20.61
Fluvastatin	5.092	5.995	8.408	13.30	8.957	29.74
Rosuvastatin	68.37	>100	67.63	–	–	–
Abiraterone acetate	4.315	2.717	9.863	5.203	22.60	5.310

### Statin, Abiraterone Acetate, and Their Combination Repressed the Migratory Ability of NB Cells

**Figure 4:**
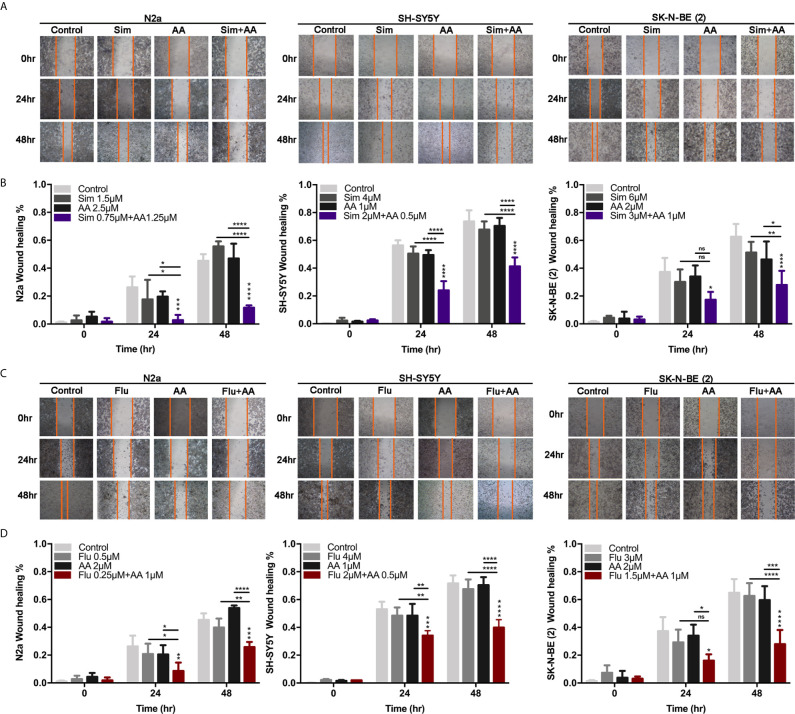
The migration of cells was attenuated by the combination of statins with AA. **(A, C)** A scratch was made in a six-well plate using a 20 μl tip, images were taken at 0, 24, and 48 h with indicated treatment in N2a, SH-SY5Y, and SK-N-BE (2). **(B, D)** Quantification of the wound healing was assessed over time using Image-Pro Plus 6.0. Results are expressed as the migration rate between the lines. Data represent an average of three independent experiments. The data are reported as mean ± SD. * p < 0.05, ** p < 0.01, *** p < 0.001, **** p < 0.0001.

Concerning that reduced cholesterol content in plasma membrane may affect cell migration, the wound healing assay was applied to measure the effect of drugs on the migratory inhibition of NB cells. The migratory ability of neuroblastoma cells was largely reduced by combined treatment of statin and AA (p < 0.0001, **Figures 4A–D**). Whereas the migration of cells with either one statin or AA treatment at 24 or 48 h did not show significant difference from that of cells in the control group, with an exception that the migration of N2a cells treated with 1.5 μM simvastatin at 48 h was significantly higher than that of the control group (P < 0.05) (**Figure 4B**). It suggested the combination of a statin and abiraterone acetate, even at half doses, can significantly decrease NB cell migration.

### Statins and Abiraterone Acetate Reduced AR, SCAP, and SREBPs Expression *In Vitro*

Our previous study suggested that NB cells may produce androgens for the growth of themselves (6). Here the production of hormones of cultured SH-SY5Y cells in comparison with HepG2 cells were determined with LC-MS/MS analysis. Interestingly, the testosterone level of culture supernatant of SH-SY5Y cells was nearly four times that of HepG2 cells (**Supplementary Figure S4A**) after culturing for 72 h in hormone free culture medium. The difference of gene expression of a series of enzymes playing key roles in cholesterol, testosterone, and dihydrotestosterone (DHT) synthesis was determined using SH-SY5Y and HepG2 cells in normal culture condition or steroid-deprivation culture condition. Compensatory elevated expression of these enzymes on transcriptional level was observed in SH-SY5Y cells treated with culture medium supplied with 5% charcoal-stripped FBS (cFBS) in contrast to normal culture condition, for example, mRNA of HMGCR, CYP11A1, an enzyme producing pregnenolone from cholesterol, CYP17A1 and SRD5A1, an enzyme producing dihydrotestosterone (DHT) from testosterone, dramatically increased due to cFBS culturing (**Supplementary Figure S4B**). The findings above proved that NB cells produced androgens for the need of growth.

**Figure 5:**
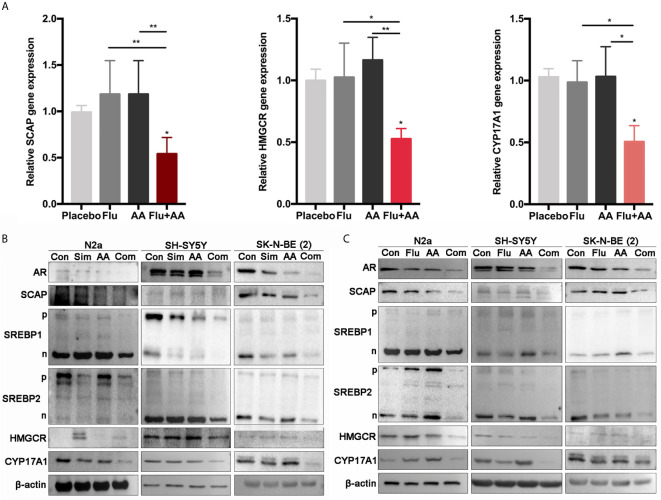
The combination of drugs significantly reduced the expression of AR and related genes. **(A)** Quantitative gene expression of SCAP, HMGCR, and CYP17A1. SK-N-BE (2) cells treated with Flu or/and AA for 24 h (the concentrations of drugs were as the same as that in **Figure 4**) to detect the expression of genes. GraphPad two-way analysis of variance (ANOVA) was used to analyze the expression differences of related genes. **(B, C)** Western blot assay (30 μg total protein per lane) was used to detect the expression of related proteins. **(B)** Simvastatin had significant effects on N2a and SH-SY5Y at 48 h, and SK-N-BE (2) had significant changes at 24 h. **(C)** Fluvastatin significantly reduced relative protein expression at 48 h. The data are reported as mean ± SD. * p < 0.05, ** p< 0.01.

We postulated that the combination of HMGCR and CYP17A1 inhibitors might have a synergistic effect on lowering the cholesterol synthesis, the subsequent testosterone production, and then reducing the activity of AR as a transcription factor. So, the expression of AR and related proteins was determined by RT-RCR and WB assays in three NB cell lines treated with the inhibitor(s) alone or combined for 24 h. Initially, as a target gene of AR, the mRNA level of SCAP dramatically decreased in SK-N-BE (2) cells treated with combined inhibitors compared with either fluvastatin or AA alone (**Figure 5A**). More than that, target gene (HMGCR and CYP17A1) expressions of SREBP1&2 were also detected. All genes’ expressions were significantly reduced under the presence of combination of drugs (**Figure 5A**). In consistent with the results on transcription level, the expression of related proteins in combination treatment group sharply decreased when compared with that in control group. In SH-SY5Y cells, although protein level of SCAP was not obviously altered when treated with a statin, AA or their combination, protein level of CYP17A1, HMGCR, and AR markedly decreased (**Figures 5B, C**). The expressions of SREBP precursors and mature forms (SREBP-p and SREBP-n, respectively, in **Figures 5B, C**) were not significantly affected by a single drug treatment, but markedly decreased due to combined treatment. These results further prove that the combination of drugs can better inhibit the growth of NB than single treatment.

### Usage of Simvastatin and Abiraterone in Nude Mice Xenograph Models, a Mini Review

Although the IC50 of fluvastatin was lower than that of simvastatin when treating SH-SY5Y cells (**Figure 3B** and **Table 1**), combined with 0.5 μM AA (1/5 of IC50), 2 μM Simvastatin (1/5 of IC50) or 2 μM Fluvastatin (1/3 of IC50) reached the inhibition rate of 50% (P < 0.001). So, simvastatin showed better synergistic effect than fluvastatin with AA (**Figure 3**). Then, documented xenographic experiments with nude mice were investigated focusing on the dosages and the inhibitory activities of simvastatin and AA (**Table 2** and **Supplementary Table S2**). As is shown in **Supplementary Table S2**, the dosages of simvastatin were 0.5–200 mg/kg/day for oral gavage, 1.25–40 mg/kg/day for intradermal injection, 5–10 mg/kg/day for intraviral injection, and 2.5–11 mg/kg/day for subcutaneous injection, respectively (**Table 3**). The concentration of AA for oral treatment was 7.5–285.7 mg/kg/day, whereas 3.5–200 mg/kg/day for intraperitoneal injection. Based on an overall consideration, we planned to orally treat the mice with simvastatin (20 mg/kg/day) or AA (150 mg/kg/day) as single treatment and the mixture of simvastatin (10 mg/kg/day) with AA (75 mg/kg/day) as the combined treatment in the following experiment.

**Table 2:** The concentration of simvastatin used *in vivo*.

Cancers	Cell lines	Simvastatin Concentration	Medication Method	Results ^a^
Glioma	U87MG	2.5 mg/kg/day**^b^**	s.c.	Positive
Human glioblastoma multiforme	GL-26 cells	1.0 mg/kg/day or 10.0 mg/kg/day	oral	Negative
Pulmonary Lymphangioleiomyomatosis	TSC2-null cells from the primary tumors	100.0 mg/kg/day	oral	Positive
Lung Carcinoma	HLMC cells from the primary patients	50.0 mg/kg/day	oral	Positive
Lung Adenocarcinoma	A549 cells	10.0 mg/kg/day	i.v	Positive
Gastric Cancer	SNU-5 cells	2.1 mg/kg/day**^b^**	i.p.	Positive
Hepatocellular Carcinoma	HepG2 cells	40.0 mg/kg/day	i.p.	Positive
Pancreatic Cancer	CAPAN-2 cells	0.5 mg/kg/day	oral	Positive
Pancreatic Ductal Adenocarcinoma	Panc-1 cells from the primary tumors	20.0 mg/kg/day	i.p.	Positive
Colorectal Cancer	SW 480 cells	6.0 mg/kg/day	oral	Positive
Colorectal Cancer	HCT116 or HT29 cells	50.0 mg/kg/day	oral	Positive
Colorectal Cancer	HCT116 p53 **^+/+^** or p53 **^-/-^**cells	20.0 mg/kg/day	i.p.	Positive
Renal Cancer	Caki-1-staR cells	6.7 mg/kg/day**^b^**	i.p.	Negative
Renal Cancer	A498 cells	5.0 mg/kg/day	oral	Positive
Prostate Cancer	22RV1 cells	25.0 mg/kg/day	i.p.	Positive
Prostate Cancer	LAPC-4 cells	11.0 mg/kg/day **^b^**	s.c.	Negative
Prostate Cancer	PC-3 cells	4.0 mg/kg/day	i.p.	Positive
Prostate Cancer	PC-3 cells	2.0 mg/kg/day or 4.0 mg/kg/day	i.p.	Positive
Prostate Cancer	PC-3 cells	2.1 mg/kg/day****or 21.0 mg/kg/day	i.p.	Positive
Breast Cancer	MDA-MB-231 cells	10.0 mg/kg/day	oral	Positive
Breast Cancer	MDA-MB-231 cells	5.0 mg/kg/day	unknown **^c^**	Positive
Breast Cancer	MDA-MB-231 cells	200.0 mg/kg/day	oral	Positive
Breast Cancer	MDA-MB-231 cells	5.0 mg/kg/day	i.v.	Positive
Osteosarcoma	KHOS or NP cells	10.0 mg/kg **^c^**	unknown **^c^**	Positive
Pediatric Acute Lymphoblastic Leukemia	specimens of T-ALL patients	20.0 mg/kg/day	oral	Negative
Chronic Myelogenous Leukemia	K562 cells	1.3 mg/kg/day ^2^ or 2.0 mg/kg/day **^b^**	i.p.	Positive
Chronic Myelogenous Leukemia	K562 cells	7.1 mg/kg/day ^2^ or14.3 mg/kg/day**^b^**	i.p.	Positive

^a^Positive: Simvastatin alone inhibited the progression of cancer; Negative: Simvastatin alone could not inhibit the progression of cancer. ^b^Concentration was converted into the dosage per kilogram per day. ^c^Relevant information was not provided in the article. Subcutaneous injection (s.c.), Intravenous injection (i.v.), Intraperitoneal injection (i.p.).

**Table 3:** The concentration of Abiraterone Acetate *in vivo*.

Cancers	Cell lines	Abiraterone Acetate Concentration	Medication Method	Results ^a^
Prostate Cancer	PC-3 cells	98.0 mg/kg/day	oral	Positive
Prostate Cancer	22RV1 cells	200.0 mg/kg**** ^c^	oral	Negative
Prostate Cancer	LNCaP cells	3.5 mg/kg/day	i.p.	Positive
Breast Cancer	MDA-MB-453 cells	285.7 mg/kg/day**** ^b^	oral	Negative
Renal Cell Carcinoma	Caki2 cells	195.8 mg/kg/day ^b^	i.p.	Positive

^a^Positive: Abiraterone acetate alone inhibited the progression of cancer; Negative: Abiraterone acetate could not inhibit the progression of cancer. ^b^Concentration is converted into the dosage per kilogram per day. ^c^Relevant information is not provided in the article. Subcutaneous injection (s.c.), Intravenous injection (i.v.), Intraperitoneal injection (i.p).

### Synergy of Simvastatin and Abiraterone Acetate Against Neuroblastoma *In Vivo*

**Figure 6:**
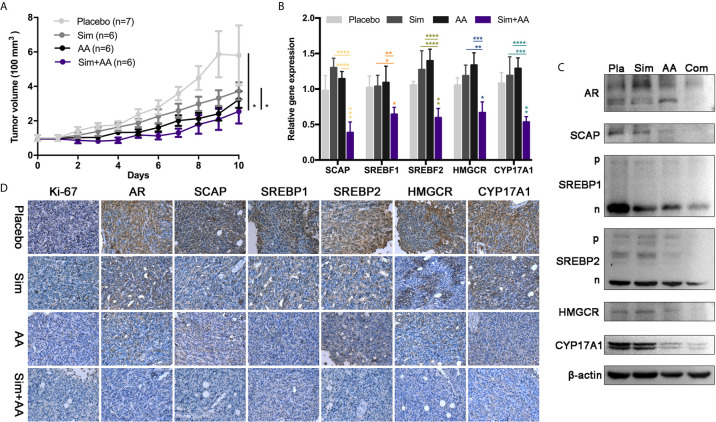
The combination of the two drugs can inhibit the growth of tumor in nude mice. **(A)** There were seven nude mice in the placebo group and six in other groups. The drug was administered, before half an hour, regularly every day. Simvastatin was 0.4 mg and Abiraterone acetate was 3 mg contained in every 100 μl suspension aid. The dosage volume was converted according to the body weight of the nude mice. There was significant difference between the two groups. Data are presented as mean ± Standard Error (SEM). **(B)** RT-PCR analysis of mRNA levels of *SCAP*, *SREBF1*, *SREBF2*, *HMGCR*, *CYP17A1*, relative mRNA levels were calibrated with β-actin. GraphPad one-way analysis of variance (ANOVA) Analysis the expression differences of related transcription genes. Data are presented as mean ± Standard Error of Mean (SEM). **(C)** Pla, placebo; Com, combination simvastatin with abiraterone acetate. Western blots showing relative protein expression in three independent experiments. **(D)** Immunohistochemistry (IHC) analysis of relative protein expression. Bar, 125 μm. *p < 0.05, **p < 0.01, ***p < 0.001, ****p < 0.0001.

Given the significant synergy demonstrated with combined simvastatin–AA treatment *in vitro*, we sought to test the efficacy of the combination strategy in neuroblastoma xenograft models. SH-SY5Y xenografts were implanted subcutaneously and allowed to grow to approximately 100 mm (3) at which point they were randomized to one of four arms for oral administration daily: vehicle, simvastatin (Sim 20 mg/kg), AA (150 mg/kg), or half-dosage combination (Sim 10 mg/kg and AA 75mg/kg). We observed tumor growth delay in all the three drug treatment groups (**Figure 6A**). It was astonishing that after 10 days of oral gavage, the mean tumor volume in the combined treatment group was about 30% of that in the control group. Further, we observed significant reduction of expressions of related genes in the combination treatment group compared with either simvastatin or AA group (**Figure 4B**). The expressions of related proteins examined by WB and IHC analysis also revealed obvious inhibition with combination treatment (**Figures 6B, C**). IHC staining intensity of Ki-67, a marker reflected the proliferation rate of tumor cells (29), and AR were both significantly weaker in combination treatment group than in the single treatment group or the control group (**Figure 6D**). The results suggested that combination of both drugs had a clinical potential to treat NB patients even with both reduced dosages.

## Discussion

In the present study, we analyzed the activation of the AR-SCAP-SREBPs-HMGCR/CYP17 axis in clinical samples and confirmed the specificity of this axis in NB. With the Oncomine NB dataset, we find a significant association between high expression of related genes and shortened survival independent of *MYCN*-amplification status. Then we explored the possibility of the combination of statin and AA for the treatment of NB *in vitro* and *in vivo*. Firstly, the IC50 values of three NB cell lines was determined when exposed to a statin or/and AA after 72 h, the combination of a statin and AA affected the proliferation and migration of NB cells significantly. Next, the expression of genes and proteins in statin or/and AA treated NB cells suggested that the inhibitors conjointly repressed AR-SCAP-SREBPs -HMGCR/CYP17 axis which dominated the development of NB. Further, we reviewed the publications that reported simvastatin and AA dosages *in vivo* and we observed the synergistic effect of simvastatin and AA on treatment of SH-SY5Y xenographic nude mice within 10 days of oral administration.

The origin of NB and ONB is uncertain (30). It’s generally believed that ONB originated from immature neuroendocrine cells of olfactory epithelium (31) as a tumor with neuroendocrine phenotype (32, 33). NB is also a neuroendocrine tumor (34) derived from neuroendocrine cells (35). Both ONB and NB are neuroendocrine neuroblastic tumors, but they are different (**Figure 1E**). Although RB and NB are embryonal tumors (26), NB is originated from the sympathetic nervous system origin (36). In spite of too limited ganglioneuroblastoma (GNB) specimens were acquired, the activation of this AR-SCAP-SREBPs-HMGCR/CYP17A1 axis was specific in NB distinct from other neuroendocrine or neuroblastic tumors.

We found that AR, the main regulator of prostate cancer (PCa), played a pivotal role in NB proliferation consistent with our previous findings (6). It is interesting that NB and PCa have something in common. Primarily, NB cell and PCa cells express AR, as same as intracranial neurons. More than that, androgen deprivation therapy (ADT) will transform PCa into castration resistant PCa (CRPC), and CRPC eventually appears neuroendocrine differentiation (NED) (37, 38). Neuroendocrine cells may come from stem/progenitor cells or neuroendocrine-like cells of primary PCa, or they may differentiate into neuroendocrine-like cells (39), moreover, anti-androgen therapy enhanced PCa NED. Although the relationship between neuroendocrine tumor and PCa remains to be elucidated, people have decades of clinical experience of PCa treatment, that will help us confront AR positive NBs.

**Figure 7:**
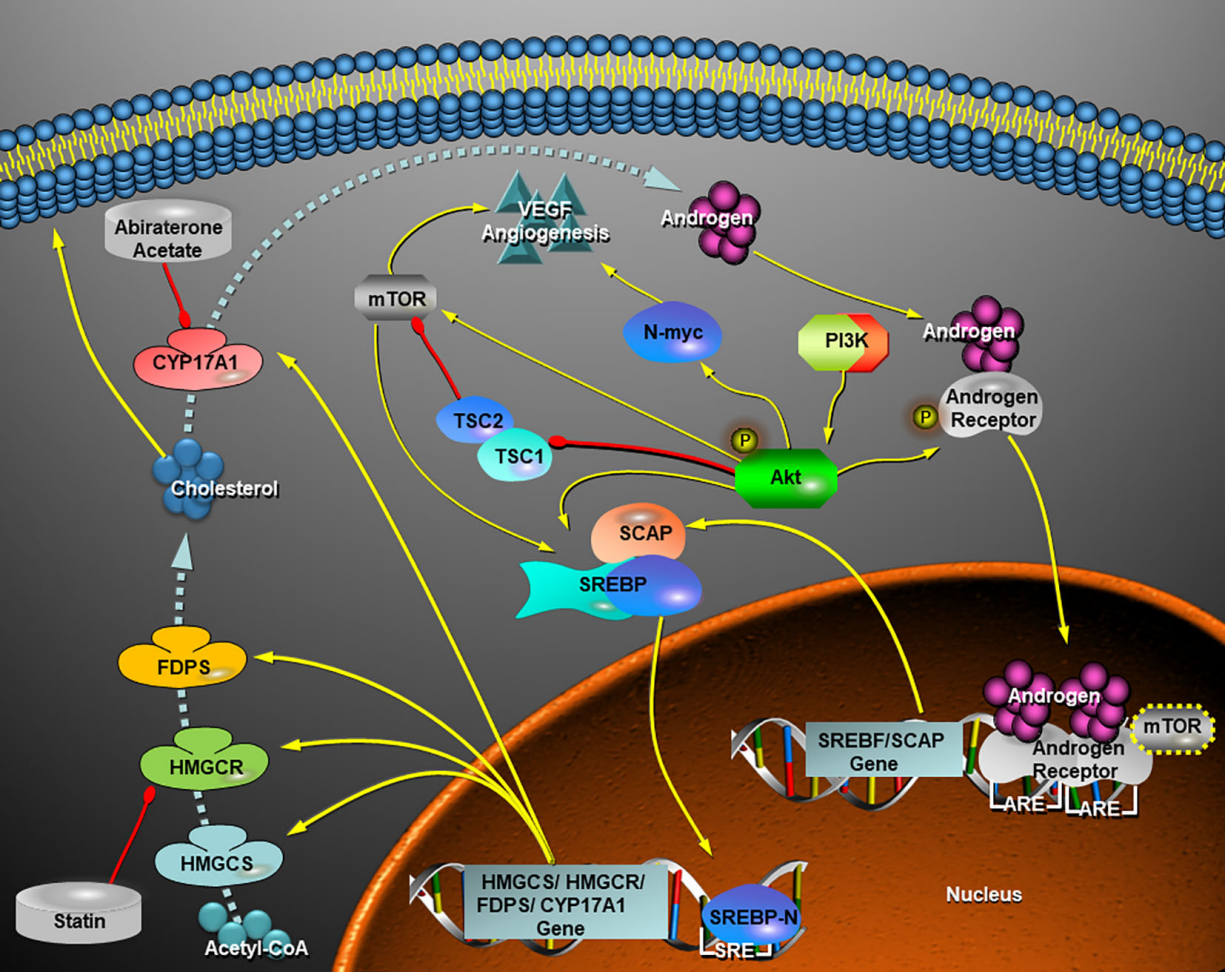
Schematic view of a hypothesis that the crosstalk of AR signaling pathway with PI3K-Akt-mTOR pathway facilitate *de novo* synthesis of cholesterol *via* activation of SREBPs and thereafter the androgen (testosterone) production increases in a neuroblastoma cell. In view of this hypothesis, a statin in cooperation with abiraterone acetate inhibits cholesterol and androgen production and therefore they turn on a repression of AR-SCAP-SREBPs-HMGCR-Cholosterol-CYP17A1-Androgen positive feedback loop. VEGF-mediated angiogenesis in *MYCN*-amplified NB cells depends on PI3K/Akt-driven signaling that most likely bypasses mTOR (43), while inhibition of AR signaling by combined statins with AA is likely independent of MYCN status. Yellow arrow, activation, induction, up-regulation, or directions of movements; red drumstick, inhibition; dashed blue arrow, synthetic route of cholesterol and androgen under the control of key enzymes. HMGCS, HMGCR, FDPS, and CYP17A1, all of them are transcriptionally regulated by SREBP-n (N-terminal of SREBPs); SRE, sterol response element; ARE, androgen receptor response element.

In our study, MYCN-non-amplified human neuroblastoma cells, SH-SY5Y, were quite sensitive to the HMGCR and CYP17A1 inhibitors *in vitro* and *in vivo*, whereas SK-N-BE (2), a MYCN-amplified human neuroblastoma cell line, were also suppressed by the two inhibitors in combination *in vitro*. The results suggested AR might be a potential target for NB therapy. In clinical treatment of NB, androgen signaling pathway has not been concerned, although targeting PI3K-Akt-mTOR pathway appears as the mainstream therapy (40). Rapamycin and its analogues as inhibitors of mTOR, such as temsirolimus, everolimus, and ridafor, show limited therapeutic effect clinically (41) maybe due to the remaining mTORC2 activity in some cases (42) or MYCN amplification (43) in others. Therefore, it is suggested targeting AKT may be a better strategy to inhibit PI3K/Akt/mTOR pathway (40). In addition, PI3K-Akt-mTOR pathway also contributes to the activation of SREBPs (**Figure 7**). Du et al. proved that Akt participated in escorting SCAP to activate SREBP2 (44), and Porstmann et al. testified that Akt activated SREBP1 by inducing glucose uptake (45). Otherwise, Audet-Walsh et al. reported AR-mTOR complex regulating SREBF1 activity in prostate cancer (12), which linked AKT-mTOR pathway together with AR signaling pathway (**Figure 7**). Therefore, we speculated that AR pathway may be associated with PI3K/Akt/mTOR pathway (**Figure 7**), and one PI3K/Akt inhibitor combined with one statin and AA would be promising in the treatment of NB.

In a word, the combination of statins and AA can achieve dual-targeted treatment of neuroblastoma with probably reduced respective side effect (toxicity). Targeting AR-SCAP-SREBPs-HMGCR/CYP17A1 axis will provide a novel strategy for neuroblastoma treatment.

## Conclusions

The combination of the two drugs, statin and AA, although in half doses, significantly suppressed the proliferation and migration ability of NB cells bypassing the regulation of MYCN, also decreased the expression of related genes and proteins. Xenograph models and clinical specimens further demonstrated that the AR-SCAP-SREBPs-CYP17/HMGCR axis promoted NB progression. Therefore, HMGCR and CYP17A1 may become potential therapeutic targets for NB.

## Data Availability Statement

The original contributions presented in the study are included in the article/**Supplementary Material**. Further inquiries can be directed to the corresponding authors.

## Ethics Statement

Ethical review and approval was not required for the study on human participants in accordance with the local legislation and institutional requirements. Written informed consent from the participants’ legal guardian/next of kin was not required to participate in this study in accordance with the national legislation and the institutional requirements. The animal study was reviewed and approved by Dalian Medical University Animal Care and Use Committee. Written informed consent was obtained from the owners for the participation of their animals in this study.

## Author Contributions

Conceptualization, YaW. Data curation, ZH, YuW, TC, and RX. Methodology, YuW. Validation, YuW, ZH, and CC. Formal analysis, TC. Investigation, JT. Resources, ZH and CC. Data curation, RX. Writing—original draft preparation, YuW. Writing—review and editing, YaW. Visualization, YuW. Supervision, YX and YaW. Project administration, YaW. Funding acquisition, ZH, CC, CN, YX, and YaW. All authors contributed to the article and approved the submitted version.

## Funding

This research was funded by the National Natural Science Foundation of China: 81172180 to YX and 81872060 to YaW; Science and Technology Project Grant from Anhui Province: 1508085QHI84 to CC, 1606c08235 and 1604a0802069 to CN; Fundamental Research Fund for Central University: WK9110000032 to CC; and Scientific Research Fund of Liaoning Provincial Education Department: LZ2019028 to ZH.

## Conflict of Interest

The authors declare that the research was conducted in the absence of any commercial or financial relationships that could be construed as a potential conflict of interest.

## Abbreviations

NB, Neuroblastoma; AR, androgen receptor; N2am Neuro2a cells; SREBPs, sterol regulatory element binding proteins; HMGCR, 3-hydroxy-3-methylglutaryl-coenzyme A reductase; PCa, prostate cancer; CYP17A1, Cytochrome P450 17A1; AA, Abiraterone acetate; mCRPC, metastatic castration resistant prostate cancer; s.c., subcutaneous injection; i.v., intravenous injection; i.p., intraperitoneal injection; OD, optical density; Sim, simvastatin; Com, combination group; IHC, immunohistochemistry; GNB, ganglioneuroblastoma; ONB, olfactory neuroblastoma; RB, retinoblastoma.
