# Supplementary material for: Synergistic Effect of Statins and Abiraterone Acetate on the Growth Inhibition of Neuroblastoma via Targeting Androgen Receptor
Source: Front Oncol. 2021 May 10;11:595285. doi: 10.3389/fonc.2021.595285 (PMC8141582; doi:10.3389/fonc.2021.595285)
Supplement: Supplementary file 1 [file DataSheet_1.docx]

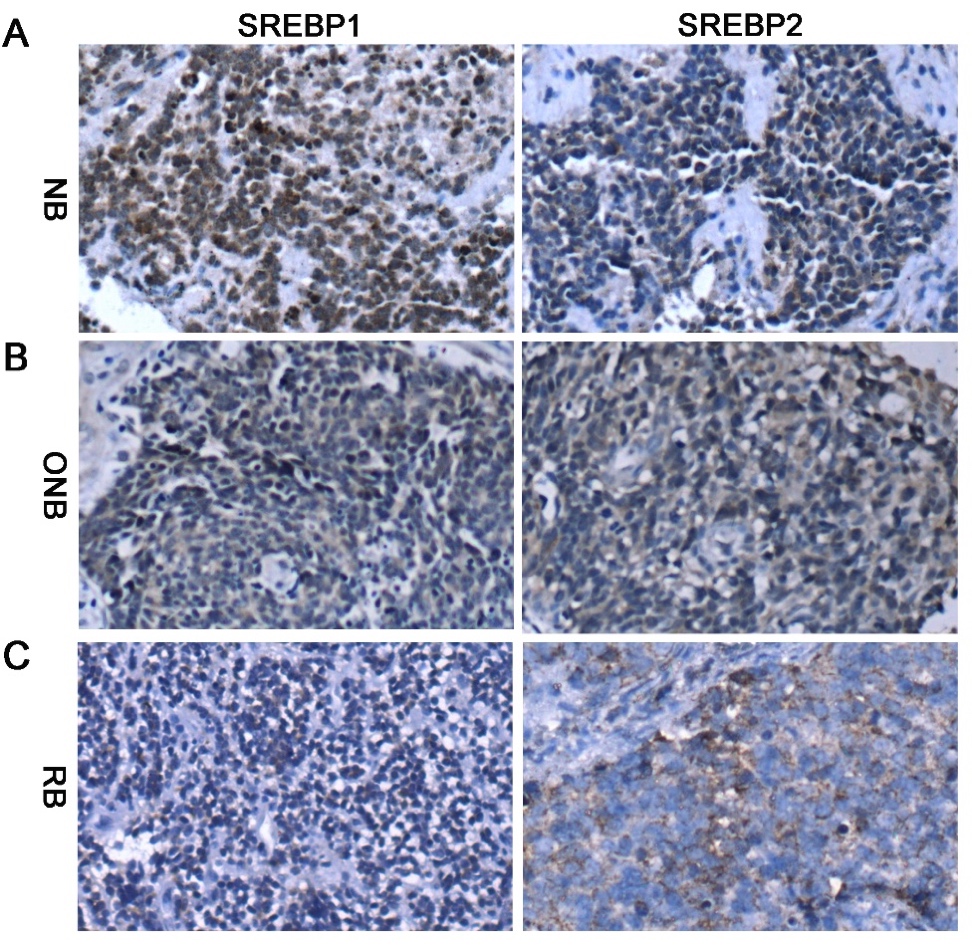


**Figure** S1. Subcellular localization of SREBPs on IHC stained clinical samples.


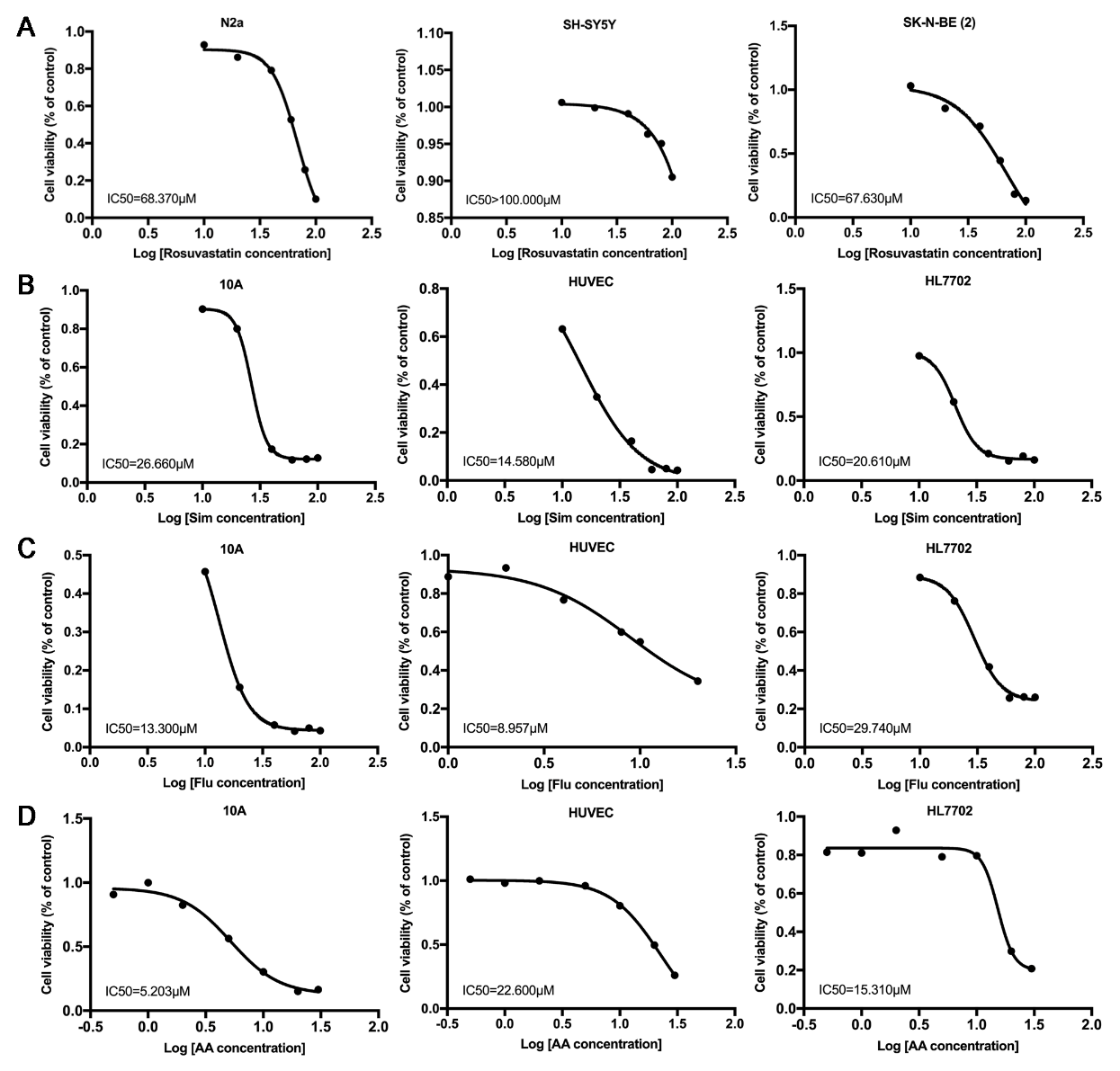


**Figure S2.** The IC50 values of statin or abiraterone acetate treatment in three NB and normal cell lines. **(A)** The effect of rosuvastatin on the proliferation of neuroblastoma. Rosuvastatin was applied to three NB cell lines with concentration gradient of 0, 10, 20, 40, 60, 80, 100 μM. **(B-D)** The IC50 of three normal cell lines with statin or Abiraterone acetate. The concentration gradient of statins was 0, 1.0, 2.0, 4.0, 8.0, 10, 20 μM, and that of AA in ethanol was 0, 0.5, 1.0, 2.0, 5.0, 10, 20, 30 μM. **(A-D)** the medium was refreshed every 24 hours, and the cell viability was detected by MTT after 72 hours treatment. IC50 of the cell was calculated by GraphPad.


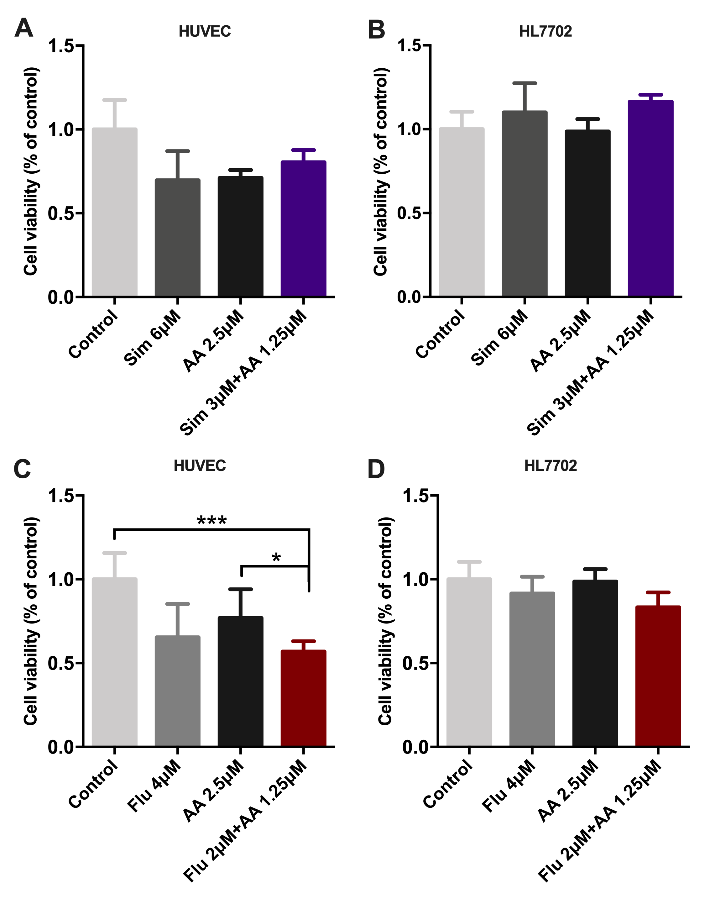


**Figure S3.** The effect of drugs combination on human non-cancer cells. In the control group, 1 ‰ DMSO and 1 ‰ ethanol was added to the culture medium, 1 ‰ ethanol was added to the culture medium with statins alone, and 1 ‰ DMSO was added to the culture medium with AA alone. The culture medium and drugs were renovated every 24 hours and the cell viability was measured using MTT method after 72 hours drug treatment. The effect of the drug combination was analyzed using GraphPad. Data are presented as mean ± standard division (SD) in three independent experiments. *p < 0.05, ***p<0.001.


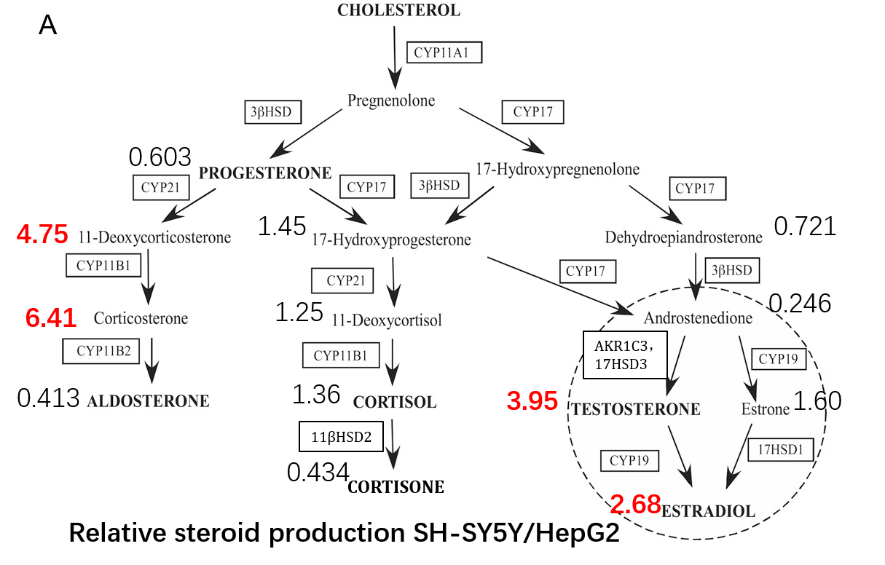

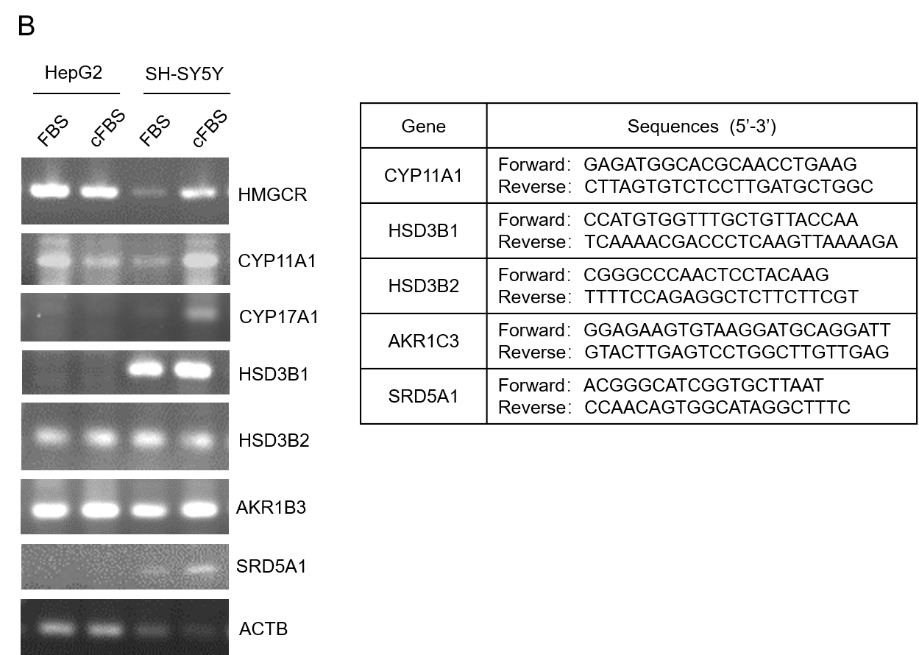


**Figure S4.** Relative steroid hormones production and enzymes expression in SH-SY5Y cells. Cells were harvested 72h after 5% charcoal-stripped FBS (cFBS) or 5% FBS (in DMEM or EMEM) culturing for gene expressing analysis using PCR method, while the culture medium was centrifuged and the supernatant was then analyzed for quantification of 13 steroid hormones. (A) Relative steroid hormones production from cholesterol in SH-SY5Y cell and HepG2 cells. The hormone producing ratio of SH-SY5Y cells to HepG2 cells was calculated and marked in red if the ratio is over 2.5. (B) cFBS culturing increased the expression of androgen-synthesizing genes in SH-SY5Y cells but not in HepG2 cells. PCR primers used in this were listed in right panel. The function of these enzymes were shown in (A). ACTB was the internal control.

**Table S1**. The index weight coefficient and 95% confidence interval of 6 genes.


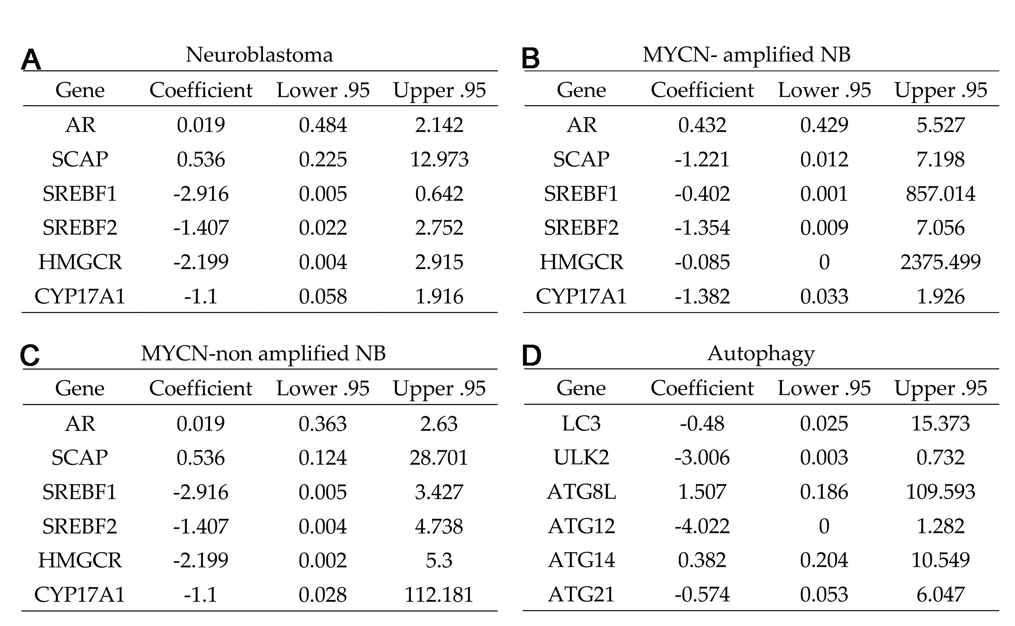


| **Cancers** | **Cell lines** | **Simvastatin Concentration** | **Medication Method** | **Results ^a^** |
| --- | --- | --- | --- | --- |
| Glioma | U87MG | 2.5 mg/kg/day ^b^ | s.c. | Positive ^[1]^ |
| Human glioblastoma multiforme | GL-26 cells | 1.0 mg/kg/day or 10.0 mg/kg/day | oral | Negative ^[2]^ |
| Pulmonary Lymphangioleiomyomatosis | TSC2-null cells from the primary tumors | 100.0 mg/kg/day | oral | Positive ^[3]^ |
| Lung Carcinoma | HLMC cells from the primary patients | 50.0 mg/kg/day | oral | Positive ^[4]^ |
| Lung Adenocarcinoma | A549 cells | 10.0 mg/kg/day | i.v | Positive ^[5]^ |
| Gastric Cancer | SNU-5 cells | 2.1 mg/kg/day ^b^ | i.p. | Positive ^[6]^ |
| Hepatocellular Carcinoma | HepG2 cells | 40.0 mg/kg/day | i.p. | Positive ^[7]^ |
| Pancreatic Cancer | CAPAN-2 cells | 0.5 mg/kg/day | oral | Positive ^[8]^ |
| Pancreatic Ductal Adenocarcinoma | Panc-1 cells from the primary tumors | 20.0 mg/kg/day | i.p. | Positive ^[9]^ |
| Colorectal Cancer | SW 480 cells | 6.0 mg/kg/day | oral | Positive ^[10]^ |
| Colorectal Cancer | HCT116 or HT29 cells | 50.0 mg/kg/day | oral | Positive ^[11]^ |
| Colorectal Cancer | HCT116 p53 **^+/+^** or p53 **^-/-^** cells | 20.0 mg/kg/day | i.p. | Positive ^[12]^ |
| Renal Cancer | Caki-1-staR cells | 6.7 mg/kg/day ^b^ | i.p. | Negative ^[13]^ |
| Renal Cancer | A498 cells | 5.0 mg/kg/day | oral | Positive ^[14]^ |
| Prostate Cancer | 22RV1 cells | 25.0 mg/kg/day | i.p. | Positive ^[15]^ |
| Prostate Cancer | LAPC-4 cells | 11.0 mg/kg/day **^b^** | s.c. | Negative ^[16]^ |
| Prostate Cancer | PC-3 cells | 4.0 mg/kg/day | i.p. | Positive ^[17]^ |
| Prostate Cancer | PC-3 cells | 2.0 mg/kg/day or 4.0 mg/kg/day | i.p. | Positive ^[18]^ |
| Prostate Cancer | PC-3 cells | 2.1 mg/kg/day or 21.0 mg/kg/day | i.p. | Positive ^[19]^ |
| Breast Cancer | MDA-MB-231 cells | 10.0 mg/kg/day | oral | Positive ^[20]^ |
| Breast Cancer | MDA-MB-231 cells | 5.0 mg/kg/day | unknown ^c^ | Positive ^[21]^ |
| Breast Cancer | MDA-MB-231 cells | 200.0 mg/kg/day | oral | Positive ^[22]^ |
| Breast Cancer | MDA-MB-231 cells | 5.0 mg/kg/day | i.v. | Positive ^[23]^ |

**Table S2.** The concentration of simvastatin used *in vivo*

**Table S2.** Continued

| **Cancers** | **Cells** | **Simvastatin Concentration** | **Medication Method** | **Results ^a^** |
| --- | --- | --- | --- | --- |

| Osteosarcoma | KHOS or NP cells | 10.0 mg/kg ^b^ | unknown ^c^ | Positive ^[24]^ |
| --- | --- | --- | --- | --- |
| Pediatric Acute Lymphoblastic Leukemia | specimens of T-ALL patients | 20.0 mg/kg/day | oral | Negative ^[25]^ |
| Chronic Myelogenous Leukemia | K562 cells | 1.3 mg/kg/day ^2^ or 2.0 mg/kg/day ^b^ | i.p. | Positive ^[26]^ |
| Chronic Myelogenous Leukemia | K562 cells | 7.1 mg/kg/day ^2^ or14.3 mg/kg/day ^2^ | i.p. | Positive ^[27]^ |

^a, Positive: Simvastatin alone inhibited the progression of cancer; Negative: Simvastatin alone could not inhibit the progression of cancer. b, Concentration was converted into the dosage per kilogram per day. c. Relevant information was not provided in the article. Subcutaneous injection (s.c.), Intravenous injection (i.v.), Intraperitoneal injection (i.p.)^

**Table S3.** The concentration of Abiraterone Acetate *in* *vivo*

| **Cancers** | **Cell lines** | **Abiraterone Acetate Concentration** | **Medication Method** | **Results** **^a^** |
| --- | --- | --- | --- | --- |
| Prostate Cancer | PC-3 cells | 98.0 mg/kg/day | oral | Positive ^[28]^ |
| Prostate Cancer | 22RV1 cells | 200.0 mg/kg ^c^ | oral | Negative ^[29]^ |
| Prostate Cancer | LNCaP cells | 3.5 mg/kg/day | i.p. | Positive ^[30]^ |
| Breast Cancer | MDA-MB-453 cells | 285.7 mg/kg/day ^b^ | oral | Negative ^[31]^ |
| Renal Cell Carcinoma | Caki2 cells | 195.8 mg/kg/day ^b^ | i.p. | Positive ^[32]^ |

^a, Positive: Abiraterone acetate alone inhibited the progression of cancer; Negative: Abiraterone acetate could not inhibit the progression of cancer. b, Concentration is converted into the dosage per kilogram per day.c. Relevant information is not provided in the article. Subcutaneous injection (s.c.), Intravenous injection (i.v.), Intraperitoneal injection (i.p)^

References in Table S2&S3

1. Kikuchi, T.; Nagata, Y.; Abe, T. In vitro and in vivo antiproliferative effects of simvastatin, an HMG-CoA reductase inhibitor, on human glioma cells. *J Neurooncol* **1997**, *34*, 233-239, doi:10.1023/a:1005753523949.

2. Bababeygy, S.R.; Polevaya, N.V.; Youssef, S.; Sun, A.; Xiong, A.; Prugpichailers, T.; Veeravagu, A.; Hou, L.C.; Steinman, L.; Tse, V. HMG-CoA reductase inhibition causes increased necrosis and apoptosis in an in vivo mouse glioblastoma multiforme model. *Anticancer Res* **2009**, *29*, 4901-4908.

3. Goncharova, E.A.; Goncharov, D.A.; Fehrenbach, M.; Khavin, I.; Ducka, B.; Hino, O.; Colby, T.V.; Merrilees, M.J.; Haczku, A.; Albelda, S.M., et al. Prevention of alveolar destruction and airspace enlargement in a mouse model of pulmonary lymphangioleiomyomatosis (LAM). *Sci Transl Med* **2012**, *4*, 154ra134, doi:10.1126/scitranslmed.3003840.

4. Polo, M.; de Bravo, M.G. Simvastatin effects on a human lung carcinoma and cholesterol homeostasis of host and non-host mice. *Arch Physiol Biochem* **2001**, *109*, 435-440, doi:10.1076/apab.109.5.435.8044.

5. Liu, H.; Wang, Z.; Li, Y.; Li, W.; Chen, Y. Simvastatin prevents proliferation and bone metastases of lung adenocarcinoma in vitro and in vivo. *Neoplasma* **2013**, *60*, 240-246, doi:10.4149/neo_2013_032.

6. Manu, K.A.; Shanmugam, M.K.; Li, F.; Chen, L.; Siveen, K.S.; Ahn, K.S.; Kumar, A.P.; Sethi, G. Simvastatin sensitizes human gastric cancer xenograft in nude mice to capecitabine by suppressing nuclear factor-kappa B-regulated gene products. *J Mol Med (Berl)* **2014**, *92*, 267-276, doi:10.1007/s00109-013-1095-0.

7. Wang, S.T.; Ho, H.J.; Lin, J.T.; Shieh, J.J.; Wu, C.Y. Simvastatin-induced cell cycle arrest through inhibition of STAT3/SKP2 axis and activation of AMPK to promote p27 and p21 accumulation in hepatocellular carcinoma cells. *Cell Death Dis* **2017**, *8*, e2626, doi:10.1038/cddis.2016.472.

8. Gbelcova, H.; Lenicek, M.; Zelenka, J.; Knejzlik, Z.; Dvorakova, G.; Zadinova, M.; Pouckova, P.; Kudla, M.; Balaz, P.; Ruml, T., et al. Differences in antitumor effects of various statins on human pancreatic cancer. *Int J Cancer* **2008**, *122*, 1214-1221, doi:10.1002/ijc.23242.

9. Xian, G.; Zhao, J.; Qin, C.; Zhang, Z.; Lin, Y.; Su, Z. Simvastatin attenuates macrophage-mediated gemcitabine resistance of pancreatic ductal adenocarcinoma by regulating the TGF-beta1/Gfi-1 axis. *Cancer Lett* **2017**, *385*, 65-74, doi:10.1016/j.canlet.2016.11.006.

10. Liu, B.S.; Xia, H.W.; Zhou, S.; Liu, Q.; Tang, Q.L.; Bi, N.X.; Zhou, J.T.; Gong, Q.Y.; Nie, Y.Z.; Bi, F. Inhibition of YAP reverses primary resistance to EGFR inhibitors in colorectal cancer cells. *Oncol Rep* **2018**, *40*, 2171-2182, doi:10.3892/or.2018.6630.

11. Kodach, L.L.; Bleuming, S.A.; Peppelenbosch, M.P.; Hommes, D.W.; van den Brink, G.R.; Hardwick, J.C. The effect of statins in colorectal cancer is mediated through the bone morphogenetic protein pathway. *Gastroenterology* **2007**, *133*, 1272-1281, doi:10.1053/j.gastro.2007.08.021.

12. Lee, J.Y.; Kim, M.S.; Ju, J.E.; Lee, M.S.; Chung, N.; Jeong, Y.K. Simvastatin enhances the radiosensitivity of p53deficient cells via inhibition of mouse double minute 2 homolog. *Int J Oncol* **2018**, *52*, 211-218, doi:10.3892/ijo.2017.4192.

13. Nitta, T.; Koike, H.; Miyao, T.; Miyazawa, Y.; Kato, H.; Furuya, Y.; Sekine, Y.; Suzuki, K. YM155 Reverses Statin Resistance in Renal Cancer by Reducing Expression of Survivin. *Anticancer Res* **2017**, *37*, 75-80, doi:10.21873/anticanres.11291.

14. Fang, Z.; Tang, Y.; Fang, J.; Zhou, Z.; Xing, Z.; Guo, Z.; Guo, X.; Wang, W.; Jiao, W.; Xu, Z., et al. Simvastatin inhibits renal cancer cell growth and metastasis via AKT/mTOR, ERK and JAK2/STAT3 pathway. *PLoS One* **2013**, *8*, e62823, doi:10.1371/journal.pone.0062823.

15. Kong, Y.; Cheng, L.; Mao, F.; Zhang, Z.; Zhang, Y.; Farah, E.; Bosler, J.; Bai, Y.; Ahmad, N.; Kuang, S., et al. Inhibition of cholesterol biosynthesis overcomes enzalutamide resistance in castration-resistant prostate cancer (CRPC). *J Biol Chem* **2018**, *293*, 14328-14341, doi:10.1074/jbc.RA118.004442.

16. Masko, E.M.; Alfaqih, M.A.; Solomon, K.R.; Barry, W.T.; Newgard, C.B.; Muehlbauer, M.J.; Valilis, N.A.; Phillips, T.E.; Poulton, S.H.; Freedland, A.R., et al. Evidence for Feedback Regulation Following Cholesterol Lowering Therapy in a Prostate Cancer Xenograft Model. *The Prostate* **2017**, *77*, 446-457, doi:10.1002/pros.23282.

17. Goc, A.; Kochuparambil, S.T.; Al-Husein, B.; Al-Azayzih, A.; Mohammad, S.; Somanath, P.R. Simultaneous modulation of the intrinsic and extrinsic pathways by simvastatin in mediating prostate cancer cell apoptosis. *BMC Cancer* **2012**, *12*, 409, doi:10.1186/1471-2407-12-409.

18. Kochuparambil, S.T.; Al-Husein, B.; Goc, A.; Soliman, S.; Somanath, P.R. Anticancer efficacy of simvastatin on prostate cancer cells and tumor xenografts is associated with inhibition of Akt and reduced prostate-specific antigen expression. *J Pharmacol Exp Ther* **2011**, *336*, 496-505, doi:10.1124/jpet.110.174870.

19. Miyazawa, Y.; Sekine, Y.; Kato, H.; Furuya, Y.; Koike, H.; Suzuki, K. Simvastatin Up-Regulates Annexin A10 That Can Inhibit the Proliferation, Migration, and Invasion in Androgen-Independent Human Prostate Cancer Cells. *Prostate* **2017**, *77*, 337-349, doi:10.1002/pros.23273.

20. Kou, X.; Jiang, X.; Liu, H.; Wang, X.; Sun, F.; Han, J.; Fan, J.; Feng, G.; Lin, Z.; Jiang, L., et al. Simvastatin functions as a heat shock protein 90 inhibitor against triple-negative breast cancer. *Cancer Sci* **2018**, *109*, 3272-3284, doi:10.1111/cas.13748.

21. Mandal, C.C.; Ghosh-Choudhury, N.; Yoneda, T.; Choudhury, G.G.; Ghosh-Choudhury, N. Simvastatin prevents skeletal metastasis of breast cancer by an antagonistic interplay between p53 and CD44. *J Biol Chem* **2011**, *286*, 11314-11327, doi:10.1074/jbc.M110.193714.

22. Mori, S.; Chang, J.T.; Andrechek, E.R.; Potti, A.; Nevins, J.R. Utilization of genomic signatures to identify phenotype-specific drugs. *PLoS One* **2009**, *4*, e6772, doi:10.1371/journal.pone.0006772.

23. Wu, Y.; Wang, Z.; Liu, G.; Zeng, X.; Wang, X.; Gao, Y.; Jiang, L.; Shi, X.; Tao, W.; Huang, L., et al. Novel Simvastatin-Loaded Nanoparticles Based on Cholic Acid-Core Star-Shaped PLGA for Breast Cancer Treatment. *Journal of Biomedical Nanotechnology* **2015**, *11*, 1247-1260, doi:10.1166/jbn.2015.2068.

24. Li, Y.; Xian, M.; Yang, B.; Ying, M.; He, Q. Inhibition of KLF4 by Statins Reverses Adriamycin-Induced Metastasis and Cancer Stemness in Osteosarcoma Cells. *Stem Cell Reports* **2017**, *8*, 1617-1629, doi:10.1016/j.stemcr.2017.04.025.

25. Samuels, A.L.; Beesley, A.H.; Yadav, B.D.; Papa, R.A.; Sutton, R.; Anderson, D.; Marshall, G.M.; Cole, C.H.; Kees, U.R.; Lock, R.B. A pre-clinical model of resistance to induction therapy in pediatric acute lymphoblastic leukemia. *Blood Cancer J* **2014**, *4*, e232, doi:10.1038/bcj.2014.52.

26. Yang, Y.C.; Huang, W.F.; Chuan, L.M.; Xiao, D.W.; Zeng, Y.L.; Zhou, D.A.; Xu, G.Q.; Liu, W.; Huang, B.; Hu, Q. In vitro and in vivo study of cell growth inhibition of simvastatin on chronic myelogenous leukemia cells. *Chemotherapy* **2008**, *54*, 438-446, doi:10.1159/000158663.

27. Oh, B.; Kim, T.Y.; Min, H.J.; Kim, M.; Kang, M.S.; Huh, J.Y.; Kim, Y.; Lee, D.S. Synergistic killing effect of imatinib and simvastatin on imatinib-resistant chronic myelogenous leukemia cells. *Anticancer Drugs* **2013**, *24*, 20-31, doi:10.1097/CAD.0b013e32835a0fbd.

28. Nesbitt, H.; Worthington, J.; Errington, R.J.; Patterson, L.H.; Smith, P.J.; McKeown, S.R.; McKenna, D.J. The unidirectional hypoxia-activated prodrug OCT1002 inhibits growth and vascular development in castrate-resistant prostate tumors. *Prostate* **2017**, *77*, 1539-1547, doi:10.1002/pros.23434.

29. Liu, C.; Armstrong, C.M.; Lou, W.; Lombard, A.; Evans, C.P.; Gao, A.C. Inhibition of AKR1C3 Activation Overcomes Resistance to Abiraterone in Advanced Prostate Cancer. *Mol Cancer Ther* **2017**, *16*, 35-44, doi:10.1158/1535-7163.MCT-16-0186.

30. Yi, X.; Zhang, J.; Yan, F.; Lu, Z.; Huang, J.; Pan, C.; Yuan, J.; Zheng, W.; Zhang, K.; Wei, D., et al. Synthesis of IR-780 dye-conjugated abiraterone for prostate cancer imaging and therapy. *Int J Oncol* **2016**, *49*, 1911-1920, doi:10.3892/ijo.2016.3693.

31. Grellety, T.; Callens, C.; Richard, E.; Briaux, A.; Velasco, V.; Pulido, M.; Goncalves, A.; Gestraud, P.; MacGrogan, G.; Bonnefoi, H., et al. Enhancing Abiraterone Acetate Efficacy in Androgen Receptor-positive Triple-negative Breast Cancer: Chk1 as a Potential Target. *Clin Cancer Res* **2019**, *25*, 856-867, doi:10.1158/1078-0432.CCR-18-1469.

32. Lee, G.T.; Han, C.S.; Kwon, Y.S.; Patel, R.; Modi, P.K.; Kwon, S.J.; Faiena, I.; Patel, N.; Singer, E.A.; Ahn, H.J., et al. Intracrine androgen biosynthesis in renal cell carcinoma. *Br J Cancer* **2017**, *116*, 937-943, doi:10.1038/bjc.2017.42.
